# Visual Search Asymmetry Due to the Relative Magnitude Represented by Number Symbols

**DOI:** 10.3390/vision5030042

**Published:** 2021-09-17

**Authors:** Benjamin A. Motz, Robert L. Goldstone, Thomas A. Busey, Richard W. Prather

**Affiliations:** 1 Department of Psychological and Brain Sciences and Cognitive Science Program, Indiana University, Bloomington, IN 47405, USA; rgoldsto@indiana.edu (R.L.G.); busey@indiana.edu (T.A.B.); 2 College of Education, University of Maryland, College Park, MD 20742, USA; prather1@umd.edu

**Keywords:** visual search, numerical cognition, quantity representation

## Abstract

In visual search tasks, physically large target stimuli are more easily identified among small distractors than are small targets among large distractors. The present study extends this finding by presenting preliminary evidence of a new search asymmetry: stimuli that *symbolically* represent larger magnitude are identified more easily among featurally equivalent distractors that represent smaller magnitude. Participants performed a visual search task using line-segment digits representing the numbers 2 and 5, and the numbers 6 and 9, as well as comparable non-numeric control stimuli. In three experiments, we found that search times are faster when the target is a digit that represents a larger magnitude than the distractor, although this pattern was not evident in one additional experiment. The results provide suggestive evidence that the magnitude of a number symbol can affect perceptual comparisons between number symbols, and that the semantic meaning of a target stimulus can systematically affect visual search.

The common orthographic forms that represent single-digit numbers (e.g., 2, 5, 6, 9) are arbitrary notational symbols. The visual appearances of these individual digits do not convey any meaning about the quantities that they represent, unlike other number systems that indicate quantities by an ordered series of rods or dots (such as Roman or Mayan systems; [1,2]). For this reason, our abstraction of numerical quantities from number symbols requires us to learn mappings between symbolic digits and the numerosities that they represent. This convention-based, symbolic relationship between numerical digits and quantities has raised an interesting question for psychologists: Does the presentation of a number symbol automatically activate some sense of the quantity it represents in a way that is measurable in a visual attention task?

Support for an automatic association between number symbols and quantities comes primarily from studies in which perceptual judgments about number symbols are affected by their numerical meaning in systematic ways. For example, when trying to identify the physically larger of two number symbols (varying in font size), performance is facilitated when the physically larger digit is numerically larger (compared to when it is numerically smaller; [3,4,5]), consistent with the effect of redundant magnitude information on non-symbolic numerical discrimination [6]. This has become known as the size congruity effect. Additionally, in perceptual comparison tasks, participants are faster and more accurate at comparing two number symbols when the quantity difference between the two digits is large (e.g., comparing 5 and 1) than when it is small (e.g., comparing 5 and 6), a classic phenomenon known as the numeric distance effect [7]. Taken together, these studies suggest that some representation of numerical quantity is activated early during the visual perception of number symbols, and that when comparing two symbols, the difference between their represented quantities modulates task difficulty (for a review, see [8]).

However, there is also reason to believe that these magnitude effects are not due to quantity *per se*, but also the visual features of the numeric stimuli. More recent studies have demonstrated that number symbol comparison effects can be explained by the physical dissimilarity of comparison digits, rather than just the quantity difference between them [9,10,11,12]. The ideal test of automatic quantity activation during numeric symbol comparison would involve judgments between digits that are composed of equivalent visual features. Conveniently, such comparisons are possible using the line-segment forms of the numbers 2 and 5 and the numbers 6 and 9. These number pairs have equivalent visual features, differing only in their orientation. However, traditional numeric distance paradigms should find no difference when making comparisons within each of these pairs (comparing 2 and 5 and comparing 6 and 9), because the numeric distance between members of both pairs is 3.

A recent examination of the size congruity effect using these line-segment forms found evidence that the same interactions between physical symbol size and numeric magnitude persist with these closely equivalent symbols [13]. Specifically, participants were faster to find a physically larger number symbol when that symbol represented a larger numeric magnitude, even when the symbol’s visual features are nearly identical to the distractors. It would seem, then, that the perception of numeric magnitude directly guides visual search processes, but it should be emphasized that this finding is an interaction and not a main effect. It is still possible that numeric magnitude only moderates the effect of physical size during visual search, possibly relatively late in the perceptual process [14]. In addition, if both physical largeness and numeric magnitude are treated as “large” on a cross-dimensional small-to-large continuum [15], then the improved performance when they are paired together could be due to a general advantage of congruent over incongruent dimension pairs. It would be ideal, then, to test the effect of numeric magnitude on visual search in a paradigm where the stimuli are nearly equivalent line-segment forms, and where there is no manipulation of the target’s physical size.

In the current study, we use an asymmetric visual search paradigm to investigate automatic magnitude effects activated during number symbol comparison. It has previously been shown that the physical size of a target visual stimulus can affect the time required to find a target among distractors. Specifically, it is easier to find a physically large target among small distractors than a small target among large distractors [16,17]. It has also been shown that random dot patterns with larger numbers of dots are found more easily among smaller sets of dots than the other way around [18]. Can similar magnitude effects in visual search be observed with number stimuli that symbolically represent different magnitudes, but have comparable visual features? Under the assumptions that (1) targets are more easily detected when they have larger, as opposed to smaller, magnitude than the distractors; and (2) magnitude representations are included in the featural representations that govern perceptual comparisons, we predict that finding a 5 among 2s and a 9 among 6s should be faster than the converse searches.

## 1. Experiment 1

### 1.1. Method

#### 1.1.1. Participants

Twelve undergraduate students from Indiana University volunteered for one credit toward their Introductory Psychology experiment participation requirement. They were 18–21 years old (average = 19.0), had normal or corrected-to-normal vision, and all self-reported to be right-handed.

#### 1.1.2. Materials

**Figure 1:**
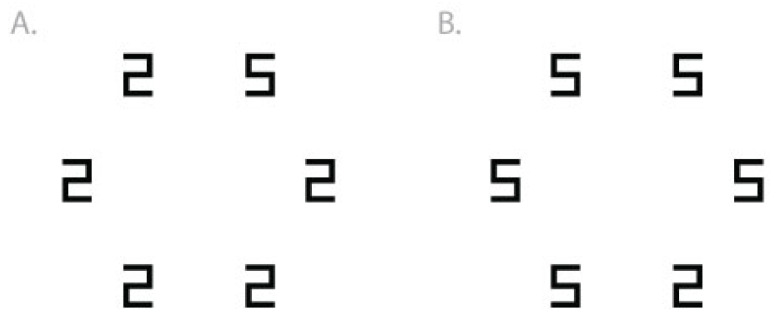
Examples of search arrays in Experiment 1. The task is to respond whether the target is present or absent in the set. (**A**) Target is the digit 5 (present at the 1 o’clock location) among 2 distractors. (**B**) Target is the digit 2 (present at the 5 o’clock location) among 5 distractors. During target-absent trials (not shown), the distractor is present at all locations.

The numbers 2, 5, 6, and 9 were rendered with black line segments on a white background. The number 2 was a horizontal mirror image of the number 5, and the number 6 was a 180° rotation of the number 9. On the monitors used in the present study, at a comfortable viewing distance, these symbols were approximately 0.6° wide and 0.9° tall. During each trial, stimuli appeared at each of six possible positions in a circular array, 5° in diameter, as shown in Figure 1. The experiment was conducted in Matlab using the Psychophysics Toolbox extensions [19].

#### 1.1.3. Procedure

Participants were seated at quiet individual computer stalls, told that they would search for a particular number (the target) among other numbers (distractors), and were asked to respond whether the target was present or absent on the screen (by pressing J or F on the keyboard, respectively), as quickly and as accurately as possible. Each trial began with a centrally located fixation point (two small concentric circles; 0.3°) presented for 1 s, and then the fixation point disappeared and the search set appeared. The target was present on 50% of trials, randomly intermixed, and during these target-present trials, the target’s location was randomized between the six possible positions (see Figure 1). During target-absent trials, the distractor was present at all six positions. The search set remained on the screen until the participant’s key press, or until 2 s elapsed, when the trial would time out.

There were two stimulus sets (2/5 and 6/9), and in each of these sets, there were two search arrangements, yielding four possible conditions (finding a 2 among 5s, finding a 5 among 2s, finding a 6 among 9s, and finding a 9 among 6s). Each of these four conditions was performed twice, for eight total blocks in the experiment. Each block began with instructions on what the target and distractor would be, then 30 practice trials followed by 80 experiment trials. Feedback was only given during practice trials. The order of the eight blocks was randomized separately for each participant, and short breaks were given between blocks. The entire experiment took about 40 min.

### 1.2. Results and Discussion

Analysis of variance (ANOVA) was used to analyze average response times for each participant, only including correct responses, using IBM SPSS. The ANOVA was a 2 × 2 × two repeated-measures design, contrasting target presence (target-absent vs. target-present), target size (numerically larger vs. numerically smaller), and stimulus set (2/5 vs. 6/9); all *p*-values reported throughout the current study are two-way. 

There was a significant main effect of target presence, as visual search tended to be slower for target-absent than target-present trials, *F*(1,11) = 11.198, *p* = 0.007, *η*_p_^2^ = 0.504. There was also a significant main effect of the target’s relative magnitude; searches for a larger numerical target (5 or 9) among smaller distractors (2 or 6, respectively) were approximately 45 ms faster overall than searches for numerically smaller target among larger distractors, *F*(1,11) = 11.975, *p* = 0.005, *η*_p_^2^ = 0.521. The difference between stimulus sets (2/5 and 6/9) did not reach statistical significance (*p* = 0.052), nor were there any significant interactions between target presence, stimulus set, and relative target size.

During experiment trials (excluding practice), the average error rate was 3.7%. Analysis with the same ANOVA design found no significant differences in accuracy between the four conditions (*p* = 0.123).

**Figure 2:**
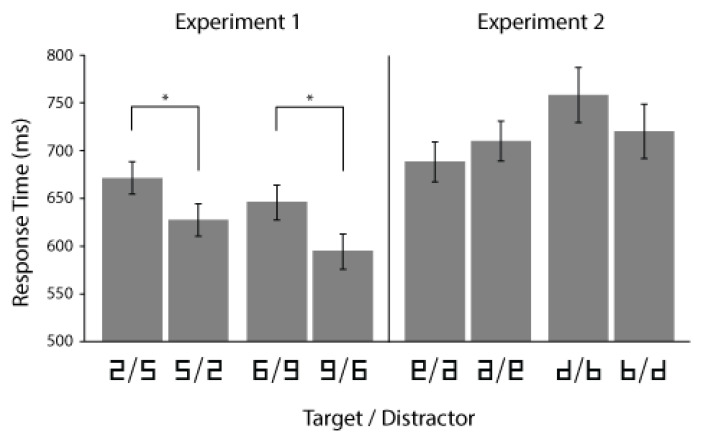
Average response times for correct responses during target-present trials in Experiments 1 and 2. Error bars indicate 95% within-subject confidence intervals; asterisks indicate *p* < 0.05.

Responses were faster when the target was a larger number than the distractor. Planned comparisons for target-present trials within each stimulus pair found that this was true for both finding 5 among 2 s (faster than 2 among 5 s; *t*(11) = 2.546, *p* = 0.027) and finding 9 among 6 s (faster than 6 among 9 s; *t*(11) = 2.744, *p* = 0.019), as illustrated in Figure 2. This search asymmetry extends previous work demonstrating that judgments about the physical size of number symbols can be affected by the numerical magnitude represented by the number symbol.

Although the target and distractor in the present search tasks are featurally equivalent to one another, there are differences in the orientation of these symbols. Before accepting that the digits’ magnitudes affected visual search times, we must examine the possibility that the stimulus orientation could yield these results.

Furthermore, there is a potential confound regarding the relative frequency of these numbers. Benford observed that the leading digits of real-life numbers (street addresses, populations, etc.) do not follow a uniform distribution [20]. Small digits more commonly lead these figures, and the frequency of occurrence declines logarithmically with increasing digits. Previous studies have shown that searching for an unfamiliar object among familiar objects is more efficient than searching for a familiar object among unfamiliar objects [21,22,23]. A generalization of this pattern might predict that, if larger numbers are less common, they would be easier to find among distractors consisting of smaller numbers than vice versa, and the observed search asymmetry may stem from familiarity differences between the target and distractors.

## 2. Experiment 2

Experiment 2 evaluated the possibility that the previous experiment’s observed search asymmetry might be caused by differences related to the orientation or the relative familiarity of the stimuli. Two new stimulus pairs were created, illustrated in the right panel of Figure 2, none representing numerical quantities. Within each of these pairs, the stimuli were either 180° rotations or mirror images of each other. Thus, if the orientations of stimuli were sufficient to cause the search asymmetries observed in Experiment 1, we might expect to replicate these effects using rotations of non-numeric stimuli. Furthermore, one of these pairs was explicitly designed to resemble the lower-case letters “b” and “d”, which differ significantly in their relative frequency in the English language (“d” is more than twice as frequent as “b”; [24]). Thus, if the frequency of common orthographic forms caused the search asymmetries observed in Experiment 1, participants would also be expected to more quickly identify “b” among “d” than the inverse.

### 2.1. Method

#### 2.1.1. Participants

Fourteen undergraduate students from Indiana University volunteered to participate for course credit. They were 18–24 years old (average = 19.9), had normal or corrected-to-normal vision, and all self-reported to be right-handed.

#### 2.1.2. Materials and Procedure

Stimuli were again rendered as black line segments on a white background, as illustrated in the right panel of Figure 2, and were the same height and width as in Experiment 1. One pair was mirror images of the digits 6 and 9, and the other pair resembled the lower case letters “b” and “d.” The procedure was the same as in Experiment 1, except that the stimuli were not described as numbers; instead participants were instructed to search for letters (in the case of “b” and “d” stimuli) and symbols (in the case of flipped 6 and 9 stimuli).

### 2.2. Results and Discussion

The average error rate was 5.4%. We conducted pairwise within-subject t-tests and found no significant differences in accuracy between flipped 6 and 9 stimuli (*p* = 0.293) or between “b” and “d” stimuli (*p* = 0.388).

Furthermore, pairwise within-subject t-tests on response times (looking only at target-present trials with correct responses) found no significant differences in response times between the two stimulus pairs. Neither a search asymmetry between flipped 6 and 9 stimuli (*p* = 0.332) nor between “b” and “d” stimuli (*p* = 0.214) was observed. While these null results do not provide positive confirmation of symbolic magnitude effects on visual search, they nevertheless suggest that differences in search times observed in Experiment 1 cannot be explained simply by differences in the orientation or the frequency of natural occurrence of comparable stimuli.

Taken together, the results of Experiments 1 and 2 provide evidence that the numeric magnitude represented by number symbols affects visual search. When the target stimulus symbolically represents a larger quantity than the distractors, participants can identify the presence of the target more easily than when the target is numerically smaller than the distractors. As is typical in visual search experiments, we observed low error rates, and the absence of differences in accuracy between stimulus arrangements suggests that numeric magnitude does not affect participants’ decision criteria, but rather, affects stimulus processing. There are two prominent explanations for this effect: either digits representing numerically larger numbers are identified more quickly because they are more salient than numerically smaller digits; or digits representing smaller numbers are more quickly rejected during search for the target.

## 3. Experiment 3

The goal of Experiment 3 was to evaluate whether the search asymmetry observed in Experiment 1 was due to increased salience of the symbolically larger target, or instead due to quick rejection of symbolically smaller distractors. These two possibilities can be distinguished by estimating the trend lines of increasing response times as more items are added to the search array, for the different search sets (finding a large number among smaller numbers vs. finding a small number among larger numbers). If distractor rejection processes influence the relative ease of finding a large target among small distractors, one might predict that this search asymmetry would be eliminated or reversed when the search set is small (with few distractors); namely, with small search arrays (e.g., 2 or 3 items), participants would have no advantage finding the numerically larger target, because they would be unaided by the ease of rejecting numerous distractors. Conversely, if the relative ease of finding a large target among small distractors is maintained even with small set sizes, the search asymmetry is due to increased salience of the target, and there should be no significant differences in the estimated slopes of the two search functions [23,25].

### 3.1. Method

#### 3.1.1. Participants

Twenty undergraduate students volunteered to participate for course credit. They were 19–30 years old (average = 21.1), had normal or corrected-to-normal vision, and all self-reported to be right-handed.

#### 3.1.2. Materials and Procedure

Ten participants performed the visual search task using only 2/5 stimuli, and the other ten used only 6/9 stimuli. Stimulus images and display configuration were identical to Experiment 1. Participants searched for the numerically larger target among the numerically smaller distractors, or for the smaller target among larger distractors. Each of these two conditions was performed twice in separate blocks, for a total of four experimental blocks, and the order of these blocks was counterbalanced across participants (either ABBA or BAAB). Each block began with instructions, then 30 practice trials (with feedback), followed by 100 experiment trials (without feedback).

In random order, either 2, 3, 4, or 6 items appeared in the search set during each trial, with items distributed evenly around the circular array. The target appeared as one of these items on a randomly chosen 50% of the trials.

### 3.2. Results and Discussion

The average error rate was 3.9%. An ANOVA found no significant differences in response accuracy between 2/5 stimuli or 6/9 stimuli (*p* = 0.076), between the two different block orders with which participants performed the task (*p* = 0.441), between finding symbolically larger or smaller numerical targets (*p* = 0.953), nor between the four set sizes (*p* = 0.161).

**Figure 3:**
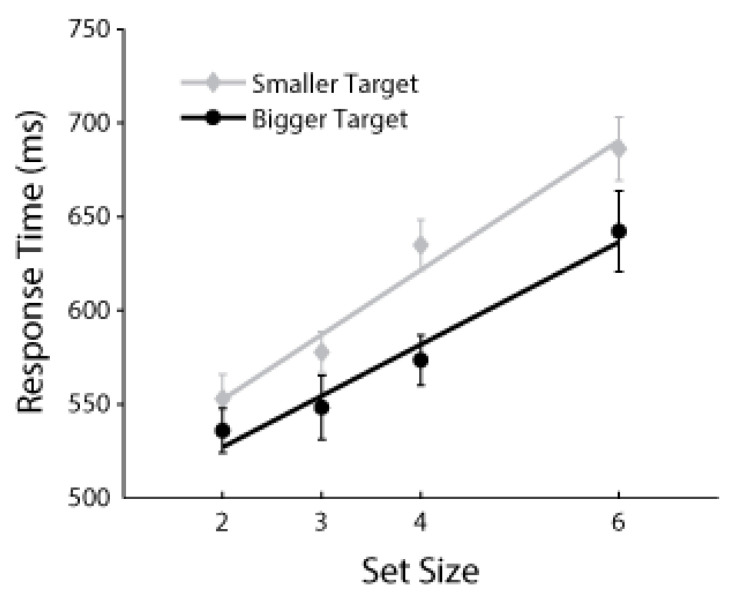
Response time during target-present trials in Experiment 3 as a function of set size. The gray markers (“Smaller Target”) combine searches for a 2 among 5s and s 6 among 9s. The black markers (“Bigger Target”) combine searches for a 5 among 2s and a 9 among 6s. The least squares linear trend line is shown for each of these conditions. Error bars indicate 95% within-subject confidence intervals.

In keeping with [25], only target-present trials were considered, because the goal of this analysis is to determine whether the quantity of distractors moderates the search asymmetry specifically during target detection. There were no significant response time differences between participants who responded to 2/5 or 6/9 stimuli (*p* = 0.744) or between block orders (*p* = 0.801). There was a main effect of set size, as increasing the number of items in the search array increased response time, *F*(3,16) = 30.287, *p* < 0.001, *η*_p_^2^ = 0.866. There was also a main effect of target size, as participants were again faster to find a numerically larger target among numerically smaller distractors, *F*(3,16) = 14.523, *p* = 0.002, *η*_p_^2^ = 0.476. The interaction between set size and target size was marginally significant, *F*(3,16) = 3.598, *p* = 0.041, *η*_p_^2^ = 0.435, but as illustrated in Figure 3, the target size effect is not completely eliminated with set sizes of 2 or 3 items. On the contrary, a post hoc within-subject paired t-test, limited only to set sizes 2 and 3, finds that participants were still faster to find larger targets than smaller targets, *t*(19) = 2.762, *p* = 0.012. For finding smaller targets, the search function increased by 34 ms per additional distractor, and for finding larger targets, the increase was 27 ms per distractor; the difference between these estimated slopes for individual subjects was not significant (*p* = 0.146).

When there were relatively few distractors (e.g., set size 2 or 3), improved task performance when finding a larger target was not eliminated, and thus, ease of rejection of numerically smaller distractors is unlikely to independently influence search performance. These results suggest that the search asymmetry between finding symbolically large over symbolically small digits stems from larger digits appearing more salient in visual search tasks. This observation is consistent with previous studies demonstrating improved performance with *physically* larger targets, an effect that is maintained across a range of set sizes (e.g., [16]).

## 4. Experiment 4

Encouraged by results thus far, we sought to replicate the findings of Experiment 3, using a procedure where each participant would perform the visual search task with both types of numeric stimuli, with a net increase in the number of trials, producing more precise estimates of the search function.

### 4.1. Method

#### 4.1.1. Participants

Fifteen undergraduate students from Indiana University volunteered for one credit toward their Introductory Psychology experiment participation requirement. Participants were recruited during the final two weeks of a spring semester and at the start of an accelerated summer term. They were 19–22 years old (average = 20.2), had normal or corrected-to-normal vision, and all self-reported to be right-handed.

#### 4.1.2. Materials and Procedure

Participants performed the task with number symbols, using 2/5 and 6/9 digit stimuli (as in Experiments 1 and 3). There were four possible search tasks (searching for a 2 among 5s, a 5 among 2s, a 6 among 9s, and a 9 among 6s), and each arrangement was performed twice in blocks, for a total of eight blocks per session. The order of blocks was randomized for each participant.

Each block began with 20 practice trials, and then had 140 experiment trials. As with Experiment 3, in random order, either 2, 3, 4, or 6 items appeared in the search set during each trial, with items distributed evenly around the circular array. The target appeared as one of these items on a randomly chosen 50% of the trials. 

### 4.2. Results and Discussion

The average error rate was 3.0%. We found no differences in accuracy due to the relative magnitude of the target number symbols (*p* = 0.780), between 2/5 and 6/9 stimulus sets (*p* = 0.280), nor between set sizes (*p* = 0.439).

**Figure 4:**
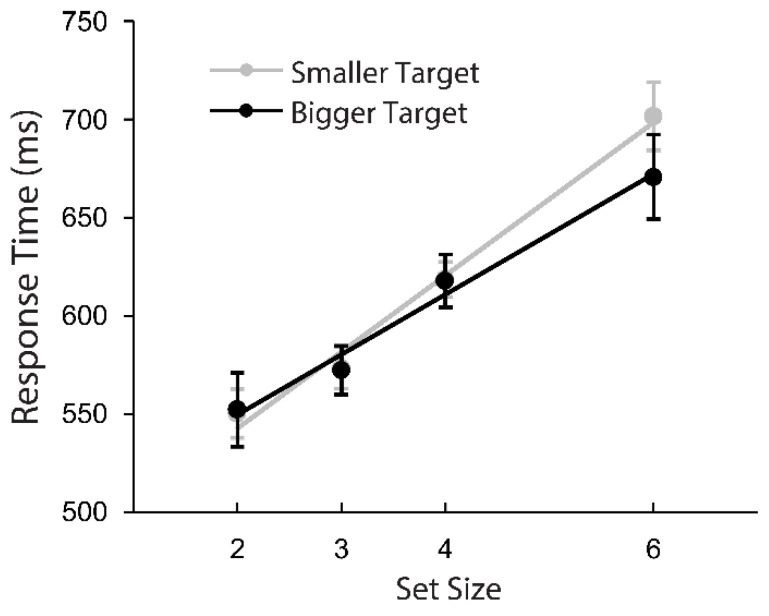
Response time during target-present trials in Experiment 4 as a function of set size. The gray markers (“Smaller Target”) combine searches for a 2 among 5s and a 6 among 9s. The black markers (“Bigger Target”) combine searches for a 5 among 2s and a 9 among 6s. The least squares linear trend line is shown for each of these conditions. Error bars indicate 95% within-subject confidence intervals.

Consistent with Experiment 3 and [25], we only analyzed response times for target-present trials. As expected, there was a main effect of set size, with more items in the search set causing increased response times, *F*(3,12) = 40.276, *p* < 0.001, *η*_p_^2^ = 0.910, and also as expected, there was no main effect of stimuli (2/5 vs. 6/9; *p* = 0.413). We also expected to find a main effect of target size on response time; however, in this experiment, the difference between finding numerically larger and numerically smaller targets was not significant (*p* = 0.599), and the interaction between set size and target size was not significant (*p* = 0.117). While error rates were comparable to Experiment 3, and set size had a robust effect on response times as expected, we saw no global evidence of participants being faster to respond to numerically larger targets than numerically smaller targets, as illustrated in Figure 4.

The overall pattern of results of Experiment 4 indicate that the search asymmetry observed in Experiments 1 and 3 may not be particularly robust. Moreover, Experiment 4 was conducted under potentially suboptimal conditions, largely during the end of a spring semester (when participants report lower levels of attentional vigilance; [26]), and with participants performing more lengthy sessions of 1280 trials, compared with 880, 880, and 520 in Experiments 1, 2, and 3, respectively.

Given the lackluster results of Experiment 4, we sought to replicate Experiment 1, using no set size manipulation, in a fifth experiment with a fresh sample.

## 5. Experiment 5

Aiming to restore confidence in the observed numeric magnitude search asymmetry, this final experiment sought to replicate the original findings of Experiment 1, without any set size manipulation.

### 5.1. Method

#### 5.1.1. Participants

Eighteen right-handed participants were recruited from the Introductory Psychology subject pool at Indiana University, and each received 1 credit toward their experiment participation requirement. Participants ranged in age from 18 to 22 (average = 19.6), and like all experiments thus far, participants had normal or corrected-to-normal vision.

#### 5.1.2. Materials and Procedure

The materials and procedure were identical to Experiment 1, except that participants performed 20 practice trials and 90 experiment trials in each block (rather than 30 practice trials and 80 experiment trials).

### 5.2. Results and Discussion

During the experiment trials (excluding practice), the average error rate was 4.1%. As in Experiment 1, there were no differences in accuracy between stimulus forms (2/5 vs. 6/9; *p* = 0.349); however, in this experiment, participants were more accurate when finding numerically larger stimuli than numerically smaller stimuli *F*(1,17) = 6.38, *p* = 0.022, *η*_p_^2^ = 0.273.

**Figure 5:**
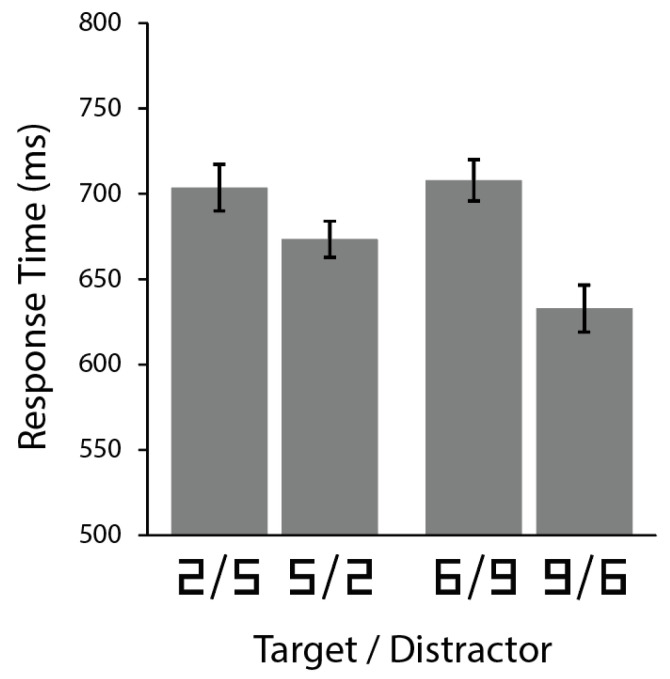
Average response times during target-present trials in Experiment 5. Error bars indicate 95% within-subject confidence intervals.

Looking only at trials where the participant responded correctly, there was a significant main effect of target presence, such that participants were faster to respond when the target was present than when it was absent, *F*(1,17) = 6.31, *p* = 0.022, *η*_p_^2^ = 0.0271. There was also a significant main effect of the numeric magnitude of the target, such that searching for the numerically larger target among smaller distractors was faster than searching for the numerically smaller target, *F*(1,17) = 9.76, *p* = 0.006, *η*_p_^2^ = 0.365. As with Experiment 1, although participants had directionally faster response times with 6/9 stimuli than 2/5 stimuli, this difference did not reach statistical significance (*p* = 0.06). This pattern of results in Experiment 5 is shown in Figure 5.

## 6. General Discussion

Using featurally equivalent visual stimuli, in three out of four experiments, participants were faster to find a numerically larger target among numerically smaller distractors, compared with finding smaller targets among larger distractors. This is the first report of this effect, but the search asymmetry described herein is also evident in the graphs included in Wang, Cavanagh, and Green [22], where plots showed that finding a 5 among 2s was roughly 50 ms faster than finding a 2 among 5s (see Figure 4 of their report, with a set size of 6), although they did not explicitly draw a comparison between these conditions. The results of Experiment 2 suggest that this effect cannot be explained only by the orientation of the visual stimuli, or the relative frequency of these stimuli in everyday experience. Instead, the visual search asymmetry seems to be due to the relative magnitudes represented by these number symbols, specifically that numerically larger targets appear more salient during visual search.

The present results are consistent with past findings that perceptual judgments about number symbols are systematically affected by the quantities that the symbols represent. Previous studies of numerosity on symbol comparison tasks, including visual search experiments (e.g., [27]), drew comparisons between markedly distinct visual stimuli, and did not control for possible confounds related to the physical similarity of comparison digits. The current experiment provides such control, using the ideal case of number symbol comparisons when the physical features of the stimuli are equivalent [13], and omitting the physical size manipulations that are typical of investigations using the size congruity effect.

However, what the present study gains in experimental control, it also lacks in generalizability, with results being limited to the isolated pairs of 2/5 and 6/9 stimuli. Thus, we see these results as providing additional empirical support for the view that numeric quantities influence perceptual comparisons, and providing a new experimental tool for measuring these influences, but we hesitate to make definitive claims on the basis of these results alone, particularly considering that the main effect was not observed in Experiment 4. Given that symbol magnitude effects during size congruity paradigms occur late in visual processing [14], it may be the case that participants who are less vigilant during extended testing sessions will not show these effects. More research, perhaps using other numeral systems (e.g., Indian Devanagari, where the symbols for 3 and 6 are horizontal mirror images), is needed to build the generalizability of these effects. Still, considering the high degree of featural comparability between the number symbols in the present study, the most parsimonious explanation is that the numeric quantity associated with these symbols influenced visual search in Experiments 1, 3, and 5.

The observation that participants were influenced by the meaning of stimuli in a visual search task also contributes to an ongoing debate about the role of learned stimulus categories in visual perception and the deployment of visual attention. Jonides and Gleitman’s [28] classic observation of search differences when the ambiguous target O (in an array of letters) was described as the letter “oh” or the number “zero” provided some initial evidence, but difficulties replicating these effects (e.g., [29,30]) have cast doubt on this study as convincingly demonstrating semantic analysis to be invoked during visual search. The current result, a visual search asymmetry due to a symbol’s magnitude-related meaning, provides new perspective on this issue. Moreover, results from Experiment 2 suggest that this search asymmetry cannot be fully explained by the symbol’s frequency in everyday experience, and thus, the present effect is distinct from the influence of familiarity on visual search performance. Instead, it is likely that dedicated brain regions for processing magnitude (e.g., [31]) may be automatically activated for symbols that are associated with a particular magnitude, and thus, they influence visual processing of these symbols.

The qualitative form of number representation elicited by number symbols in this visual search task remains unknown. While larger digits appeared more salient among smaller digits, this increased salience could be due to differences of ordinality, cardinality, or some blending of the two. Neuroanatomical correlates of number sense, as well as individual and developmental differences in number sense, could be explored using this asymmetry, and these might shed light on the conceptual form of numerosity that systematically influenced the present visual search task.

## 7. Author Note

The search asymmetry described in this study was initially discovered during undergraduate classroom activities led by author B.A.M., using a visual search applet developed by author T.A.B. with a National Science Foundation grant (#0127561) to the Mind Project. We thank the Indiana University students involved in these activities for their curiosity and their contributions, and Madalyne Pattee for her assistance with data collection.

## Data Availability

Study data and materials are available at https://osf.io/sgecb/.
